# Insight into the genetic diversity of *Mycobacterium bovis* isolated from cattle in Ghana

**DOI:** 10.1128/spectrum.02133-25

**Published:** 2026-01-27

**Authors:** David Atomanyi Barnes, Nathan Grant-Biney, Philip Tetteh, Emelia Konadu Danso, Prince Asare, Jeewan Thapa, Tamsin S. Barnes, Adwoa Asante-Poku, Samuel Adjei, Stephen V. Gordon, Yasuhiko Suzuki, Chie Nakajima

**Affiliations:** 1 Division of Bioresources, Hokkaido University International Institute for Zoonosis Control, Sapporo, Japan; 2 Noguchi Memorial Institute for Medical Research, College of Health Sciences, University of Ghana 58835, Accra, Ghana; 3 Veterinary Services Directorate, Airport, Ghana; 4 Department of Molecular Microbiology and Immunology, Bond Life Science Center, University of Missouri 14716, Columbia, Missouri, USA; 5 The University of Queensland, School of Veterinary Science 1974, Brisbane, Queensland, Australia; 6 School of Veterinary Medicine, University College Dublin 8797, Dublin, Ireland; 7 Hokkaido University Institute for Vaccine Research and Development, Hokkaido, Japan; UJF-Grenoble 1, CHU Grenoble, Grenoble, France

**Keywords:** bovine tuberculosis, Ghana, *Mycobacterium bovis*, spoligotyping, MIRU-VNTR

## Abstract

Bovine tuberculosis (bTB), caused primarily by *Mycobacterium bovis*, poses significant risks to cattle and public health in Ghana. Previous studies reported varying prevalence (3%–21%) and *M. bovis* strain distributions, necessitating a deeper understanding of the molecular epidemiology of bTB to guide control strategies. This study investigated the genetic diversity and phylogenetic relationships of *M. bovis* isolates from cattle in Ghanaian abattoirs and compared results with strains from other African countries. Spoligotyping analysis of 28 *M*. *bovis* isolates confirmed 6 distinct spoligotypes, with SB0944 being the most prevalent (64.3%). Mycobacterial Interspersed Repetitive Units–Variable Number Tandem Repeats (MIRU-VNTR) genotyping analysis further resolved the isolates into 22 genotypes, with loci MIRU26, QUB26, and ETR-A showing the highest allelic diversity. Mixed infections were found in 10% of culture-confirmed cases, indicating a high risk of co-infection with multiple strains in the area. Comparative analysis revealed that Ghanaian isolates clustered within the African 1 (Af1) clonal complex, consistent with strains from Nigeria, Burkina Faso, and Cameroon, but distinct from the BCG-like and other strains that were dominant in Algeria and, to some extent, in Mali. Minimal strain homogenization of West-Central Africa with other regions suggests localized evolution, shaped by ecological barriers, regulated transhumance, and possible competitive exclusion among strains. These findings provide evidence for the need for region-specific bTB control measures that reflect Africa’s unique livestock dynamics and micro-epidemic patterns. Enhanced surveillance with advanced genotyping methods could improve outbreak tracking and inform targeted interventions to mitigate bTB’s impact on livestock in this region.

**IMPORTANCE:**

Bovine tuberculosis (bTB), caused primarily by *Mycobacterium bovis*, is a neglected disease in many low- and middle-income countries, including Ghana. Our study reveals that while *M. bovis* strains in Ghana show high local genetic diversity, they belong almost exclusively to a single clonal complex (Af1) shared across West-Central Africa. Despite regional regulations governing transhumance and cattle movement, the observed strain mixing suggests an “island-like” evolutionary structure for *M. bovis* in this area; that is, diverse within West-Central African countries but genetically isolated. This pattern is shaped by natural barriers, such as deserts and forests, which restrict strain flow while permitting intense intra-regional transmission. Understanding *M. bovis* evolution in this context is essential for tailoring effective, region-specific interventions. Our findings highlight the need to treat West-Central Africa as a community of interconnected but genetically distinct bTB zones, calling for both local action and coordinated regional strategies to mitigate bTB.

## INTRODUCTION

Bovine tuberculosis (bTB) is a chronic bacterial disease caused primarily by *Mycobacterium bovis*. Although cattle are the principal host, the disease has also been reported in various domesticated and wild species, including antelopes, sheep, goats, wild boar, and deer (1). It is unclear how much of the global human TB case count is linked to *M. bovis*-associated zoonotic TB, and this poses a severe threat to the WHO’s “End TB 2030” aim (2).

bTB is present in most African countries, yet comprehensive data on its prevalence and impact remain limited (3). This dearth of information contributes to an ongoing zoonotic risk, posing a significant public health challenge across the continent (4, 5). In Ghana, although research on bTB is relatively scarce, available studies suggest that the disease is endemic (6–8). Findings indicate a high prevalence of bTB in cattle slaughtered at abattoirs, with 3–21% of animals displaying tuberculous lesions during postmortem examinations (8, 9). However, surveillance and research efforts face considerable challenges, primarily due to economic constraints, inadequate logistics, and insufficient infrastructure (10).

The transmission of bTB among cattle occurs through multiple pathways, including direct contact with infected animals, ingestion of contaminated feed or water, and inhalation of airborne bacteria (9, 11). In Ghana, the widespread movement of cattle and the dominance of extensive farming systems facilitate inter-herd transmission, increasing the risk of infection spread (12, 13). Similar trends have been reported in other African countries where bTB is endemic, highlighting the urgent need for coordinated regional control measures (5, 14, 15). To effectively guide control and prevention strategies, it is crucial to understand the genetic diversity of *M. bovis* in order to identify the strains circulating in cattle populations and to help elucidate transmission pathways.

bTB has been eradicated in several developed countries through test and slaughter of reactor cattle (16). However, the implementation of such control strategies in developing countries is unfeasible due to financial limitations. In these circumstances, spatial patterns, transmissions, and pathogen diversity become essential data for identifying the infection source and supporting the design of targeted control strategies, including restrictions on animal movement (17, 18).

In this study, we collected granulomatous suspected tuberculous lesions from cattle slaughtered at selected abattoirs (the major slaughter facilities) and slaughterhouses (smaller slaughter facilities located in more remote areas) in Ghana, cultured them for mycobacteria, and genotyped the isolates. Additionally, we combined the data set from Ghana (based on spoligotyping patterns and MIRU-VNTRs) to other African countries that allowed us to contextualize the *M. bovis* strains in Ghana relative to the genetic diversity of strains that exist within the West-Central African sub-region.

## MATERIALS AND METHODS

### Study areas

**Fig 1:**
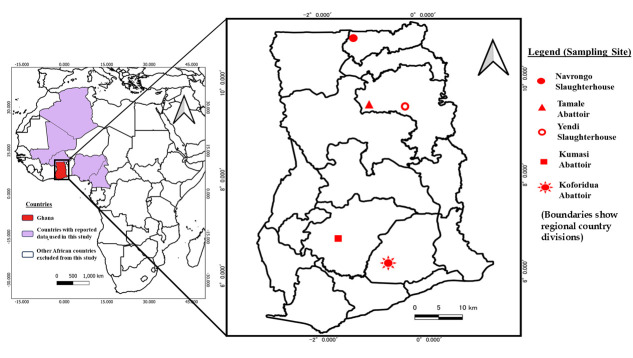
Map of Ghana showing various abattoir sampling sites. The figure was generated using QGIS 2.2 (www.qgis.org) and shp files obtained from the GADM database of Global Administrative Areas (www.gadm.org).

A cross-sectional study was conducted to collect granulomatous suspected tuberculous lesioned tissues during routine post-mortem examination of cattle at selected abattoirs and slaughterhouses across Ghana. Districts were selected based on their high cattle population densities, strategic location along major livestock trade routes, and the presence of active livestock markets. Within each district, the main abattoir or slaughterhouse was chosen based on its role as a central processing facility for animals sourced from surrounding farms and markets. These were the Navrongo slaughterhouse, Tamale abattoir, Yendi slaughterhouse, Kumasi abattoir, and Koforidua abattoir. Sample collection occurred between July and December 2023, and the sampling sites are shown in Fig. 1. Most cattle slaughtered at these facilities originate from various regions within Ghana, with additional inflows from neighboring countries such as Burkina Faso and Nigeria.

### Postmortem examination and sample collection

Permission and approval of the Veterinary Services Directorate (VSD) in Ghana were sought prior to the sampling. All slaughtered cattle were subjected to a postmortem examination during meat inspection, which involved palpating, examining, and cutting various organs (such as the lymph nodes, the lungs, liver, spleen, and heart). When multiple lesions were observed in a single animal, all were collected for documentation; however, only the most prominent or representative lesion was selected for culture. As a result, each cultured sample used in the analysis was derived from a distinct animal. Tissues showing lesions that highly suggested tuberculosis were taken out using sterile scissors and forceps and put into sterile plastic specimen Ziploc bags that were appropriately labeled. The samples were sent in Styrofoam boxes on ice packs to the University of Ghana Noguchi Memorial Institute for Medical Research (NMIMR) for storage at −30°C. The samples were frozen for one to five months prior to processing due to logistical constraints and the distance between the laboratory and the abattoir locations.

### Sample processing: decontamination, culture, and isolation

The sample decontamination and culture were as described by Acquah et al*.* (6). The samples were homogenized in a tissue grinder. For decontamination purposes, the homogenized tubercles were suspended in sterile phosphate-buffered saline (PBS) in a 50 mL conical tube. Five milliliters of every tissue homogenate suspension were decontaminated in labeled 50 mL conical tubes with an equal volume of 5% (wt/vol) oxalic acid, with intermittent vortexing at room temperature for 30 min. To pellet the mycobacteria from the suspensions, centrifugation was used for 30 min at 4,000 rpm. For inoculation onto Lowenstein-Jensen (LJ) culture media, the pellets were carefully decanted and re-suspended in 2.0 mL of PBS after the supernatant had been carefully strained. Four labeled, supplemented LJ media slants were inoculated with 100 μL of the decontaminated tissue homogenate; two of the LJ slants were supplemented with 2% glycerol, while the other two were supplemented with 0.4% sodium pyruvate. The LJ tubes were incubated at 37°C for 8 weeks, with growth observed on a weekly basis. The culture-positive isolates were collected by transferring loopfuls of bacterial colonies into sterile tubes containing Tris-EDTA buffer. The tubes were then subjected to heat inactivation at 90°C for 30 min before being transported to Hokkaido University International Institute for Zoonosis Control (HU-IIZC) for further analysis.

### DNA extraction and mycobacterial species confirmation

DNA from the heat-killed culture isolates was extracted using the method as described by Asante-Poku et al. (19). The multiplex PCR assay described by Bakshi et al. (20) and Kapalamula et al. (21) was used to confirm the isolates as *M. bovis*. The PCR reaction mixture contained 0.5 µL of 10 µM each primer targeting the region of difference (RD4), 0.8 µL of 25 mM MgCl_2_, 2 µL of 2.5 mM dNTP mix, 2 µL of 5 M betaine, 0.1 µL of 5 U/µL of GoTaq DNA polymerase (Promega Co., Madison, WI, USA), and 1 µL of template DNA. The volume of the PCR reaction was adjusted to 20 µL with double-distilled water (DDW). CBS1 (5′-TTCCGAATCCCTTGTGA-3′), which was the common forward primer, CBS2 (5′-GGAGAGCGCCGTTGTA-3′) for *M. bovis*, and CBS3 (5′-AGTCGCCGTGGCTTCTCTTTTA-3′) for *M. tuberculosis* were used as the primers for the reaction. The cycling conditions were as follows: 35 cycles of 1 min of denaturation at 96°C, 30 s of annealing at 50°C, 30 s of extension at 72°C, and a final elongation of 3 min at 72°C. The PCR products were run on 1.5% agarose gel stained with GelRed (Biotium, Inc., CA, USA) for 25 min at 100 V with an expected band size of 263 bp and 168 bp for *M. tuberculosis* and *M. bovis,* respectively.

### *M. bovis* genotyping

DNA of all confirmed *M. bovis* isolates was used for genotyping studies. The typing was performed using Spoligotyping and Mycobacterial Interspersed Repetitive Unit-Variable Number Tandem Repeat (MIRU-VNTR). Spoligotyping was performed as described by Kamerbeek et al. (22) and Solo et al. (23). Initially, a primer pair designed for the direct repeat sequence was used to amplify the direct repeat (DR) region of *M. bovis* by PCR. Subsequently, the amplified PCR products were hybridized into a specialized membrane with 43 different oligonucleotide probes, each of which matched a different spacer in the DR region. The numbers “1” and “0,” respectively, were assigned to each spacer to indicate whether spacers were present in the PCR products or not. Consequently, the spoligotyping pattern was used to generate an octal digit. This code number was then entered into the online database https://www.mbovis.org/database.php accessed on 10th April 2024, which facilitated the generation of the corresponding SB number for further analysis and comparison.

MIRU-VNTR typing using 15 loci (24) was conducted as previously described with a minor modification (Mtub29 and Mtub34 were replaced with ETR-F and QUB11a) to improve discriminatory power (23, 25). The rest of the loci used were ETR-C, QUB26, QUB11b, MIRU04, QUB3232, ETR-A, ETR-B, MIRU26, MIRU16, MIRU10, Mtub30, QUB4156, and MIRU31. An initial denaturation at 95°C for 3 min preceded 35 cycles of 95°C for 15 s, 55°C for 20 s, and 72°C for 45 s, with a final extension at 72°C for 5 min. For loci QUB11b and MIRU04, the primer annealing temperature was adjusted to 50°C. PCR products were separated by electrophoresis on a 2% agarose gel prepared in Tris-borate-EDTA (TBE) buffer. The gel was stained with ethidium bromide for 30 min with gentle shaking. After staining, the PCR products were visualized under UV light, and amplicon sizes were determined using a 50 bp DNA ladder. The results were reported as number designations, where each number represented the number of repeats detected at a particular locus.

### Allelic diversity of MIRU-VNTR loci

The allelic diversity was calculated for each of the 15 MIRU-VNTR loci. This provides an indication of the relative contribution of each locus to the overall discriminatory power of the MIRU-VNTR as a typing aid. The following formula was used to estimate the allelic diversity (*h*) for each locus:


$$h=1-\sum x_{i}^{2}\left[\frac{1}{n\left(n-1\right)}\right]$$

where *xᵢ* is the frequency of the *i*th allele at the locus, and *n* is the number of isolates analyzed. Allelic diversity values (*h*) were classified as follows: loci with *h* ≥ 0.6 were considered to have high allelic diversity, indicating substantial genetic variability; loci with 0.3 ≤ *h* < 0.6 were categorized as having moderate allelic diversity, reflecting a moderate level of variability; and loci with *h* < 0.3 were considered to have low allelic diversity, suggesting limited genetic variability (26, 27).

### Phylogenetic analysis

*Mycobacterium bovis* genotypes were defined by spoligotype number using the Mbovis.org database (https://www.mbovis.org/). The MIRU-VNTRplus online tool was used to analyze MIRU-VNTR profiles (28, 29). To investigate the genetic connections between the isolates, MIRU-VNTR data were employed. Distance matrices were calculated via the MrBayes programming tool (30), and a minimum spanning tree (MST) and an unweighted pair group average (UPGMA) tree subsequently were drawn using BioNumerics software package version 7.6 (Applied Maths, Sint-Martens-Latem, Belgium). To be considered genetically distinct, isolates had to meet a minimum threshold of one single locus variation. Clusters were identified as groups of two or more isolates that shared the same spoligotype and/or MIRU-VNTR patterns.

### Genetic variation of *M. bovis* in Ghana compared to neighboring African countries

**Fig 2:**
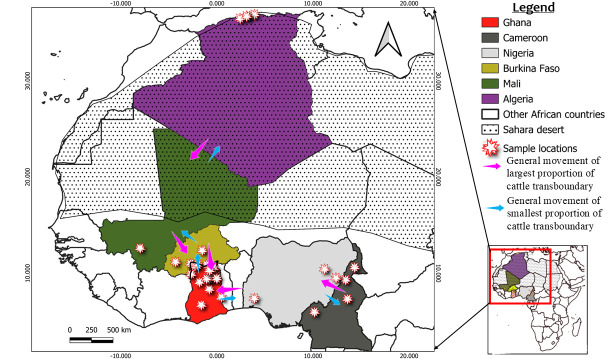
Map showing the location of the six countries and the transboundary movement of cattle across their borders based on report from ECOWAS Regional Policies and Response to Manage Pastoral Movements. The pink arrow indicates the primary direction of cattle movement across the border between two countries. The blue arrow shows the movement of the smaller proportion of cattle across the border between two countries. The figure was generated using QGIS 2.2 (www.qgis.org) and shp files obtained from the GADM database of Global Administrative Areas (www.gadm.org).

A pie chart and MST were generated to examine the genetic diversity of *M. bovis* isolates from Ghana in comparison to some nearby African countries. Figure 2 shows the relative transboundary movement of cattle across the selected West African countries, as well as bordering countries, based on the report from the Regional Policies and Response to Manage Pastoral Movements within the ECOWAS (Economic Community of West African States) Region, 2019 (31), as well as data from Egbe et al. (32) and Berger (33). In total, 552 isolates were included in the analysis, comprising 198 isolates from Ghana reported in the study by Acquah et al. (6), additional isolates from Ghana collected in this study (*n* = 28), and isolates from other African countries, such as Burkina Faso (*n* = 25) (34), Cameroon (*n* = 214) (32), Nigeria (*n* = 26) (25, 35), and Mali (*n* = 20) (36). Additionally, 41 isolates from Algeria, collected from the Mediterranean region (27), were considered for comparative purposes in the spoligotype analysis, given the evolutionary relationships between strains from North and sub-Saharan Africa. All non-African 1 (Af1) (37) strains were excluded from the downward VNTR MST analysis. Selection criteria for the isolates from the African countries included the following:

(i) Geographic origin: The isolates came from nations that bordered a west African country close to Ghana or were part of West Africa, excluding isolates from regions geographically distinct from sub-Saharan Africa for VNTR analysis.
(ii) Missing data: Out of 10 VNTR loci, isolates with no more than three missing data points were included.

Ten VNTR loci—ETR-C, QUB26, QUB11b, MIRU04, ETR-A, ETR-B, QUB11a, MIRU26, MIRU16, and Mtub30—were used to construct the network. *M. bovis* clustering rate (CR) was then estimated using the following formula:

$Clustering\;rate\;(CR)=\frac{n_{c}}{N}$, where *n*_*c*_ is the number of clustered strains and *N* is the total number of strains analyzed.

## RESULTS

A total of 69 suspected tuberculous lesion samples were analyzed from five different sampling locations. The majority of samples were from Tamale (59.4%), followed by Kumasi (17.4%), Yendi (13.0%), Navrongo (8.7%), and Koforidua (1.4%). A total of 28 *M*. *bovis* isolates were obtained from the 69 suspected tuberculous lesion samples. The distribution of suspected tuberculous samples and *M. bovis* isolates across the five locations is summarized in Table 1.

**TABLE 1:** Distribution of suspected tuberculous lesion samples and *M. bovis* isolates by location

Location	No. of suspected tuberculous lesion samples	No. of *M. bovis* isolates obtained	% of *M. bovis* isolates from suspected Tb lesion for each location
Tamale	41	17	41.5
Kumasi	12	5	41.7
Yendi	9	5	55.6
Navrongo	6	1	16.6
Koforidua	1	0	0.0
Total	69	28	40.6

### Geographical distribution and diversity of *M. bovis* spoligotypes in Ghana

All 28 isolates analyzed in this study were confirmed as *M. bovis* using the multiplex PCR assay. Spoligotyping revealed six distinct spoligotypes: SB0944, SB0300, SB0328, SB1026, SB1027, and SB2757. All 28 isolates belonged to the Af1 clonal complex, with the characteristic absence of spacer 30 (37). The distribution of the spoligotypes from this study is presented in Table 2. The distribution varied across the different locations. One isolate, SB2757, isolated from Kumasi, was distinct from the other spoligotypes, exhibiting a large spacer deletion from spacer 4 to spacer 26.

**TABLE 2:** Spoligotype analysis of the 28 Ghanaian isolates

Location in Ghana	Spoligotype designation (%)						Total (%)
	SB0300	SB0328	SB0944	SB1026	SB1027	SB2757	
Tamale	2	2	11	1	1		17
Kumasi	3		1			1	5
Yendi			5				5
Navrongo			1				1
Total (%)	5 (17.9)	2 (7.1)	18 (64.3)	1 (3.6)	1 (3.6)	1 (3.6)	28 (100)

### Association between lesional pattern, tissue type, and *M. bovis* spoligotypes

The relationship between pathological presentation and the circulating *M. bovis* strains was investigated. Analysis of lesion patterns revealed that while the predominant spoligotypes SB0944 and SB0300 were associated with a high proportion of generalized disease 66.7% and 80.0%, respectively (Table 3), this association was not statistically significant (Fisher’s exact test, *P* = 0.206). To further assess dissemination capacity, we analyzed the distribution of spoligotypes across different tissue categories (Table 4). Again, no significant statistical association was found (*P* = 0.415); however, the descriptive data were highly informative. The predominant spoligotype SB0944 demonstrated a broad tissue tropism, being isolated from all tissue categories and accounting for the majority of infections in extrapulmonary organs (5/7). Similarly, SB0300 was cultured from multiple extrapulmonary sites.

**TABLE 3:** Association between *M. bovis* spoligotypes and pathological presentation in cattle from Ghana

Spoligotype	Total isolates	Localized lesion (% of localized lesions)	Generalized lesion (% of generalized lesions)	% generalized lesions of spoligotype^*a*^
SB0944	18	6 (60.0)	12 (66.9)	66.7
SB0300	5	1 (10.0)	4 (22.2)	80.0
SB0328	2	2 (20.0)	0 (0.0)	0.0
SB1026	1	0 (0.0)	1 (5.6)	100.0
SB1027	1	1 (10.0)	0 (0.0)	0.0
SB2757	1	0 (0.0)	1 (5.6)	100.0
Total	28	10 (100.0)	18 (100.0)	64.3

^*a*^ % generalized lesions of spoligotype = (generalized lesion/total isolates of that spoligotype) × 100.

**TABLE 4:** Distribution of *Mycobacterium bovis* spoligotypes across tissue categories in cattle from Ghana

Tissue category	SB0944	SB0300	Other spoligotypes^*b*^	Total
Lungs	10	1	4	15
Lymph nodes	2	1	1	4
Trachea	1	1		2
Extrapulmonary organs^*a*^	5	2		7
Total	18	5	5	28

^*a*^ Extrapulmonary organs: kidney, spleen, liver, and mammary gland.

^*b*^ Other spoligotypes: SB0328, SB1026, SB1027, and SB2757.

### Molecular analysis based on 15 MIRU-VNTR loci

**Fig 3:**
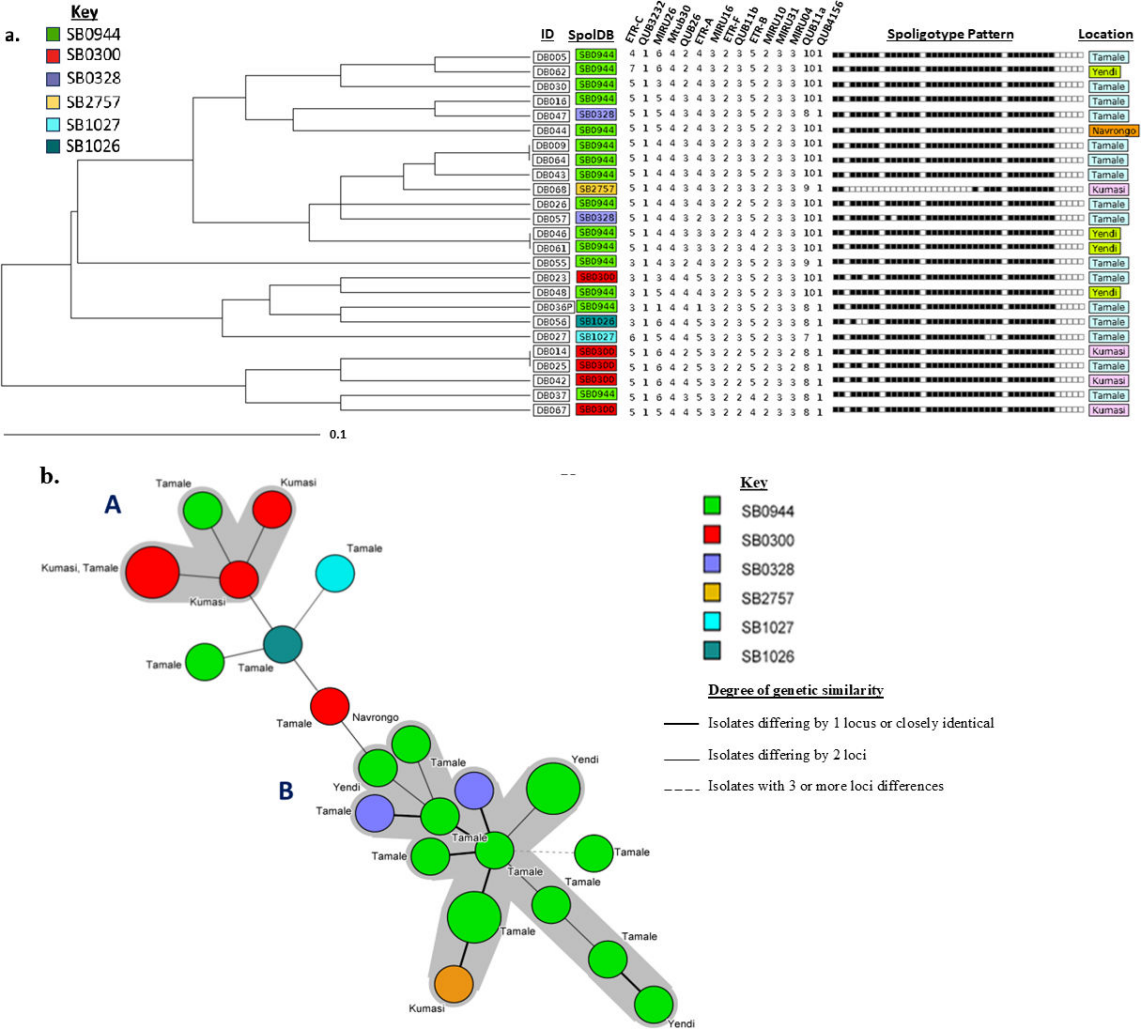
Analysis of 25 isolates based on 15 MIRU-VNTR data. (**a**) UPGMA phylogenetic tree based on 15 MIRU-VNTR analysis of 25 Ghanaian isolates. (**b**) Minimum spanning tree of 25 Ghanaian isolates based on 15 MIRU-VNTR analysis. The gray zone collectively shows all connections that span clusters A and B.

In the subsequent MIRU-VNTR analysis, three isolates, namely DB059, DB066, and DB071 (Table S1), were excluded because they proved to have mixed infections with multiple bands on several VNTR loci. Thus, 25 isolates were used for the VNTR analysis. The six spoligotype patterns identified in this study were thus further characterized into 22 distinct genotypes on inclusion of MIRU-VNTR data (Fig. 3a). The predominant spoligotypes SB0944 and SB0300 were each further differentiated into 13 and four unique genotypes, respectively. The rest of the spoligotypes were each subdivided into at most two genotypes.

The MST revealed two main groups (Fig. 3b). Group A was primarily composed of isolates from Tamale and Kumasi, with SB0944 and SB0300 as the main spoligotypes observed. Group B consisted of genotypes isolated from Yendi, Tamale, Kumasi, and Navrongo and was dominated by the SB0944 spoligotype.

### Allelic diversity

The results obtained from analyzing the MIRU-VNTR loci are presented in Table 5, which includes the information on the locus alias, observed tandem repeat numbers for each allele, allelic variants (AV), and allelic diversity (*h*) for each locus. The *h* ranging from 0 to 0.72 reflects the level of genetic diversity at each locus. Among the loci analyzed, MIRU26, QUB26, and ETR-A exhibited the highest allelic diversity with values of 0.72, 0.64, and 0.60, respectively. QUB11a, ETR-C, ETR-B, and QUB11b displayed moderate allelic diversity indices ranging between 0.3 and 0.6. On the other hand, MIRU04, MIRU31, and VN2401 demonstrated poor allelic diversity (*h <* 0.3). The remaining loci showed no genetic variation.

**TABLE 5:** Allelic diversity values across 15 MIRU-VNTR loci in 25 *M*. *bovis* isolates

Locus	Alias	Number of tandem repeats											AV^*b*^	Allelic diversity
		0	1	2	3	4	5	6	7	8	9	10		
2966	MIRU26		1		2	9	6	7					5	0.72
4052	QUB26			7	11	7							3	0.64
2165	ETR-A		1	1	2	13	8						5	0.60
a^*a*^	QUB11a								1	8	2	14	4	0.57
577	ETR-C				5	1	17	1	1				5	0.47
2461	ETR-B				3	4	18						3	0.42
2163	QUB11b			6	19								2	0.34
580	MIRU04			2	23								2	0.12
3192	MIRU31			1	24								2	0.04
2401	VN2401				1	24							2	0.04
a^*a*^	QUB3232		25										1	0
960	MIRU10			25									1	0
1644	MIRU16				25								1	0
4156	QUB4156		25										1	0
a^*a*^	ETR-F			25									1	0

^*a*^ Non-standard 24 loci by Supply et al*.* (24).

^*b*^ AV: allelic variants.

### Contextualizing the diversity of *M. bovis* isolates between Ghanaian isolates and those from neighboring countries

Table 6 shows the number of isolates from each country employed in each plot, either from this study or retrieved from literature. The data sets from Algeria and Mali were excluded in the VNTR analysis because they did not meet the inclusion criteria previously outlined.

**TABLE 6:** Total number of isolates included for each country in this study

Country	No. of isolates included in spoligotype study	No. of isolates included in MIRU-VNTR study
Ghana	226	25
Cameroon	214	208
Nigeria	26	26
Burkina Faso	25	25
Mali	20	0
Algeria	41	0
Total	552	284

**Fig 4:**
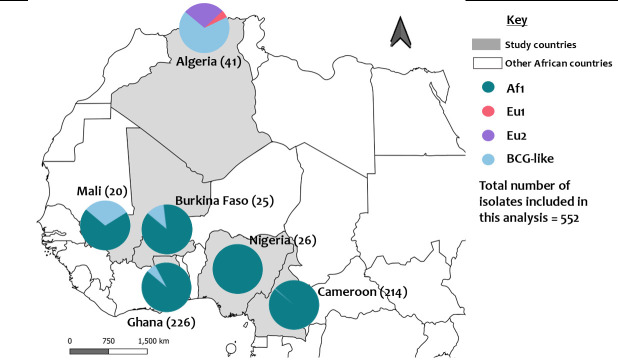
Spatial distribution of spoligotype diversity and dominance in the six countries. Numbers in brackets indicate the total number of isolates from each country included in the analysis. BCG-like: including BCG-like and others. The figure was generated using QGIS 2.2 (www.qgis.org) and shp files obtained from the GADM database of Global Administrative Areas (www.gadm.org).

The spoligotyping results revealed distinct geographic diversity in *M. bovis* clonal complexes across the six African countries (Fig. 4; Table S2). The majority of isolates from Algeria, located in Northern Africa where the sampling sites were Mediterranean, belonged to the BCG-like and European 2 (Eu2) clonal complexes (27, 38). These BCG-like isolates displayed spoligotype patterns similar to the vaccine strain BCG (3). European 1 (Eu1) and Eu2 clonal complexes, marked by the absence of spacers 11 and 21, respectively, were identified only in Algeria. There was no Af1 clonal complex detected in Algeria (27). In contrast, the Af1 clonal complex was the most prevalent in the Western-Central African countries.

In Ghana, SB0944 was predominant (42%), followed by SB0300 (14%). The majority of SB types, including SB0944 and SB0300, belonged to the Af1 clonal complex (94%), while 6.2% belonged to BCG-like and other complexes. In Cameroon, SB0944 was the most common Af1 type, followed by another Af1 type SB0953, while only a small proportion of isolates (0.5%) were classified as BCG-like (32). In Nigeria, all isolates (100%) were identified as Af1 (25, 35).

In Burkina Faso and Mali, the majority of isolates also belonged to the Af1 clonal complex, although with limited diversity. In Mali, 70% of the isolates were Af1, while 30% were non-Af1, SB0134 (36). This analysis by spoligotyping had a CR of 0.93.

**Fig 5:**
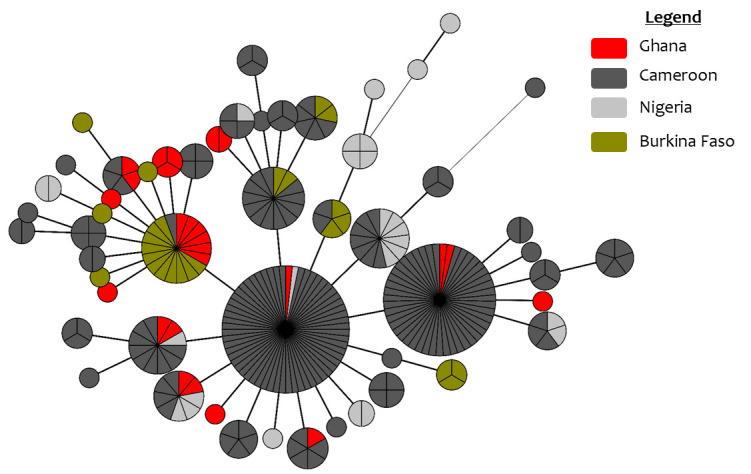
Minimum spanning tree of 284 isolates based on 10 MIRU-VNTR loci. The MST illustrates genetic relationships among isolates, with distinct clusters representing groups of closely related genotypes. Line thickness reflects the number of differing loci: thicker lines indicate fewer differences (1–2 loci), while thinner lines represent greater divergence (≥3 loci). Likewise, line length denotes genetic distance, with longer lines corresponding to more allele differences across the 10 loci. Node size: representing the number of isolates within each cluster.

Using 10 MIRU-VNTR loci, an MST was constructed based on the comprehensive data set of 284 Af1 isolates. The results revealed a close relationship between the Ghanaian isolates and the isolates from the other Western-Central African countries. The Ghanaian isolates seem to be diversely distributed, and 64% of the isolates form clusters with isolates from other countries (Fig. 5). The MIRU-VNTR analysis had a lower CR compared to spoligotyping at 0.75.

## DISCUSSION

bTB is a contagious infectious disease caused mainly by *M. bovis* that affects cattle and poses a significant public health risk. Past studies in an abattoir in Ghana conducted by Adu-Bobi et al. (39) revealed that 73.1% of carcasses with lesions suggestive of bTB were found to be acid-fast on microscopic examination, giving an indication of the potential risk of zoonotic transmission to humans through the consumption of contaminated beef. Another study conducted on dairy and research farms in Ghana reported a 2.48% prevalence of bTB in cattle, with MTBC species identified, including *Mycobacterium africanum* (40).

In this study, we aimed to investigate the molecular diversity and phylogenetic relationships of *M. bovis* isolated from cattle slaughtered across selected abattoirs in Ghana and compared them to *M. bovis* isolates from neighboring African countries for which data were available. Understanding the genetic diversity and transmission patterns of *M. bovis* in different regions is essential for developing effective bTB control strategies (41–43) and can provide valuable insights into regional variations and potential transmission sources (37).

All 28 isolates were confirmed as *M. bovis*, consistent with the findings of Acquah et al. (6), who reported that bTB in Ghana was caused primarily by the animal-adapted species *M. bovis*, with only 1% caused by the human-adapted species (*Mycobacterium africanum* and *Mycobacterium tuberculosis sensu stricto*). This contrasts with the findings of Ameni et al. (44) from Ethiopia, who reported that about 27% of isolates from grazing cattle were *Mycobacterium tuberculosis*.

Our study found different *M. bovis* spoligotypes in the selected abattoirs in Ghana, adding information to what is known about the distribution and diversity of *M. bovis* in Ghana (6, 9, 45). The spoligotyping results revealed six distinct *M. bovis* spoligotypes in Ghana, all belonging to Af1 clonal complex, with SB0944 being the most prevalent (64.3%), especially in the northern regions of Tamale, Yendi, and Navrongo. This finding is consistent with Acquah et al. (6), who also reported SB0944 as being dominant in the northern region of Ghana, comprising 37.9% of their isolates. Additionally, we found SB0300, a subclonal variant of Af1, in 17.9% of our isolates, similar to the 12.8% reported by Acquah et al*.* Our results support the dominance of the Af1 clonal complex in Ghana, reflecting patterns observed across West Africa, and highlight the absence of African 2 (Af2) clonal complex strains, which are dominantly observed in East Africa (46). While Acquah et al.(6) reported a high proportion of uncharacterized spoligotypes, we did not detect a comparable frequency of uncharacterized spoligotypes in our study.

Interestingly, the spoligotype SB2757, observed in a single isolate from Kumasi (3.57%; 1/28), has lost spacers 4–24 and 26. The presence of spacer 25 is due to its known duplication, with another spacer 25 sequence located between spacers 31 and 32 (47). SB2757 accounted for approximately 2.95% (6/54) of the spoligotypes reported by Acquah et al. (6), where it was first identified in the northern belt of the country. In contrast, our study is the first to report this spoligotype in the middle belt region, indicating a possible geographic spread or introduction of this strain into new areas.

Three isolates showed mixed infections, confirmed by the presence of multiple bands for two or more MIRU-VNTR loci, specifically QUB-11b, MIRU-26, ETR-C, QUB-26, and ETR-B (Table S1). This implies that about 10% (3/28) of the animals were infected with multiple *M. bovis* strains. A similar pattern was reported by Biffa et al. (48) in Ethiopia, where half of infected cattle carcasses they examined had multiple *M. bovis* strain*s*. The authors attributed this to factors such as the frequent movement and mingling of livestock. Mixed-strain infections have also been documented in human tuberculosis cases (49), underscoring the relevance of this observation. It is important to note that the 10% rate of mixed-strain infection reported here is likely a conservative estimate. The genotyping techniques employed may fail to detect minor strains present in low quantities within a lesion. Furthermore, as only a single lesion was cultured per animal, our study design would not identify animals harboring different strains in separate, anatomically distinct lesions.

The six spoligotype patterns identified in this study were further characterized into 22 distinct genotypes by MIRU-VNTR (Fig. 3a). This further differentiation shows the existence of considerable genetic variability within the *M. bovis* population of our study, revealing intra-spoligotype genetic diversity. This finding implies that more complex genotyping methods than spoligotyping alone, such as MIRU-VNTR, are essential to distinguish between strains with better precision, as has been reported by Ordaz‐Vázquez et al. (50). The intra-spoligotype diversity revealed by MIRU-VNTR genotyping has implications for tracking transmission pathways for bTB, providing greater resolution to aid in transmission mapping (51).

The allelic diversity observed among the MIRU-VNTR loci (Table 5) in our study further cements the genetic variability within this *M. bovis* population. In particular, MIRU26, QUB26, and ETR-A loci were found to have the highest allelic diversity. These loci were potent discriminators, indicative of a significant genetic differentiation at these sites within the *M. bovis* genome, while QUB11a, ETR-C, ETR-B, and QUB11b displayed moderate allelic diversity. Thus, these highly variable loci can be selected for genotyping to ensure high-resolution discrimination that is necessary for effective molecular epidemiological studies. These same loci were also reported by Damina et al. (25) as contributing to differentiation among isolates in Nigeria. Interestingly, QUB3232 did not show discriminatory power in Ghana, although this locus usually exhibits high allelic diversity (1, 21, 27, 50). Similar results were also reported for Af1 isolates from Burkina Faso (34). This was because the repeat number of QUB3232 was one in Af1 isolates from these countries. This finding suggests that the characteristics of locally prevalent strains should be investigated in advance when a typing method is applied in the area. The practical implications for this are its relevance in resource optimization, typing efficiency, and epidemiological tracking in resource-limited areas (32). By identifying which loci are most informative, resources can be focused on analyzing these loci rather than using a larger number that may be redundant or less informative (25, 50).

Next, our study aimed to contextualize the diversity of *M. bovis* isolates by comparing those from Ghana with data from neighboring African countries obtained from the literature (6, 25, 27, 32, 34–36). All the isolates from Ghana in this study belonged to the Af1 clonal complex (i.e., 28/28). In Nigeria, Burkina Faso, and Cameroon, molecular epidemiology studies have identified that the majority of *M. bovis* isolates also belonged to the Af1 clonal complex (3, 52). SB0944, which is the parent strain of Af1, emerged as the major common spoligotype in all the West-Central African countries (Table S2). While SB0944 was predominant across several countries, distinctions were mainly seen in the less represented spoligotypes. This suggests peripheral diversity of less common strains that may represent local adaptations or evolutionary pathways alongside dominant strains.

The spoligotyping data further revealed distinct patterns among isolates from Algeria (27) (Fig. 4), with most of the isolates belonging to the BCG-like and European clones, which had a paucity of representation in other West-Central African countries. This finding was consistent with other studies (3), with the exception of Mali, a neighboring country with which Algeria shares a border (53, 54). Like Algeria, approximately 30% (6/20) of the isolates from Mali also belonged to SB0134, which is one of the dominant spoligotypes in France and minor in other West-Central African countries (27, 36). The restricted geographic prevalence and distinct genetic diversity of the Af1 clonal complex in West-Central Africa may be attributed to natural geographic barriers, such as the Sahara Desert separating West-Central Africa from the Mediterranean region (Fig. 2) and dense forest zones acting as ecological barriers between West and East Africa. In contrast, the absence of Af2 in West-Central Africa and its presence in East Africa (46) may be influenced by historical cattle trade routes, ecological adaptations, and host population dynamics. The drier, open landscapes of East Africa, coupled with long-standing pastoralist practices and transboundary livestock movement, may have facilitated the persistence and spread of Af2 in the region, while limiting its establishment in the more forested and geographically fragmented zones of West-Central Africa. These factors likely contribute to the distinct spatial distribution of *M. bovis* clonal complexes in Africa (55), as has been shown in other studies (56).

It is interesting to note that ECOWAS is the only regional economic community in Africa with specific regulations governing transhumance (31), primarily requiring pastoralists to carry an International Transhumance Certificate (ITC), which outlines routes, herd size, and health status. Despite these regulatory efforts, the presence and movement of the Af1 clonal complex in the West and Sahel continue to spread. The Af1 clonal complex may be dispersed throughout West-Central Africa by transhumance movement of cattle; however, the unique differences in the spoligotype frequencies of the Af1 strains in the countries where Af1 is dominant (Table S2), along with their MIRU-VNTR genotypes, reveal that each of these West-Central African countries has a unique population structure. This suggests that the population of *M. bovis* in each of these countries is not being mixed sufficiently to homogenize the populations, and that some of the populations have been isolated long enough for country-specific genotypes and population structures to arise (52).

This isolation of distinct *M. bovis* populations across countries could be attributed to several factors. One possibility is the direct transfer of cattle across borders for immediate slaughter, preventing the introduction of imported *M. bovis* strains into the local herds (31). Another hypothesis that has been proposed is the potential existence of competitive exclusion among *M. bovis* clones, where established clones may hinder the establishment of newly introduced ones (1, 57). This phenomenon could have significant implications for disease control strategies. The observation of geographically localized genotypes is not limited to the Af1 clonal complex. This pattern has been consistently reported in various global studies on *M. bovis* phylogeography (3, 37, 52, 58).

The MST based on 10 MIRU-VNTR loci provided more insight on the genetic relationship among the Ghanaian isolates and those from Burkina Faso, Nigeria, and Cameroon (Fig. 5). Spoligotyping produced a high CR of 0.93, indicating limited discriminatory power. However, the 10 MIRU-VNTR loci reduced the CR to 0.75, revealing greater diversity within the isolates. Our findings are consistent with the findings of Kapalamula et al. (59) in Malawi, where a CR of 0.68 was reported using 10 loci, with two locus substitutions (MIRU-10 instead of QUB26, and MIRU-31 instead of Mtub-30, adjusted for an Eu1 dominant population). Interestingly, our analysis identified three pairs of isolates with identical VNTR patterns, one of which was collected from different regions, underscoring the utility of the 10-locus VNTR method for tracking strain transmission across geographic areas. It is worth mentioning that although the analysis based on spoligotyping and MIRU-VNTR provides some level of variability and reduced clustering in identifying related isolates, high-throughput methods, such as whole-genome sequencing (WGS), may be able to provide a more thorough understanding of transmission dynamics and provide a more comprehensive understanding of the complex patterns. However, WGS is still not feasible for regular monitoring in resource-limited areas.

It is interesting to note that the genetic diversity of *M. bovis* observed in Ghana may be influenced by wildlife reservoirs, a factor that has been documented in other African ecosystems. The African buffalo (*Syncerus caffer*) is a known maintenance host in southern and eastern Africa, with infection prevalence varying significantly between herds and regions (60, 61). In Zambia, a species of antelope known as lechwe (*Kobus leche*) has been reported as a reservoir infected with the same *M. bovis* strain as cattle (62). The persistence and spread of *M. bovis* in these wildlife populations are facilitated by social behavior, environmental contamination, and predator-prey dynamics (63), leading to spillover into numerous other species (64, 65). While the specific role of wildlife in Ghana requires investigation, it is plausible that native species, such as antelopes and warthogs, could act as reservoirs or spillover hosts. Additionally, transboundary cattle movement with neighboring countries, where different wildlife reservoirs may exist, could further contribute to the genetic diversity observed. Future studies should therefore prioritize surveying wildlife in Ghana to elucidate their role in the epidemiology of bTB.

### Conclusion

The molecular diversity and phylogenetic relationships of *M. bovis* isolates from cattle in Ghana and West-Central African countries show a complex genetic landscape. More effective surveillance is needed to understand the transmission dynamics of bTB infection both at the local level in Ghana and across the wider West-Central African region. Future studies should, therefore, concentrate on determining the variables affecting the prevalence of bTB in this area and allow control strategies to be developed that are tailored to local livestock husbandry practices. Through such approaches, intervention strategies could ultimately lessen the bTB disease burden in cattle and reduce the threat of zoonotic TB to humans.

## Data Availability

The data presented in this study are available on request from the corresponding author.

## SUPPLEMENTAL MATERIAL

Table S1 spectrum.02133-25-s0001.xlsx MIRU-VNTR genotyping profiles and demographic data of *Mycobacterium bovis* isolates from Ghana.

Table S2 spectrum.02133-25-s0002.xls Spoligotype distribution and clonal complexes of *Mycobacterium bovis* isolates across African countries included in this study.

ASM does not own the copyrights to Supplemental Material that may be linked to, or accessed through, an article. The authors have granted ASM a non-exclusive, world-wide license to publish the Supplemental Material files. Please contact the corresponding author directly for reuse.
